# Chronotype and Sleep Quality in Obesity: How Do They Change After Menopause?

**DOI:** 10.1007/s13679-022-00479-9

**Published:** 2022-09-02

**Authors:** Ludovica Verde, Luigi Barrea, Claudia Vetrani, Evelyn Frias-Toral, Sebastián Pablo Chapela, Ranil Jayawardena, Giulia de Alteriis, Annamaria Docimo, Silvia Savastano, Annamaria Colao, Giovanna Muscogiuri

**Affiliations:** 1 Centro Italiano Per La Cura E Il Benessere del Paziente Con Obesità (C.I.B.O), Dipartimento Di Medicina Clinica E Chirurgia, Unit of Endocrinology, Federico II University Medical School of Naples, Via Sergio Pansini 5, 80131 Naples, Italy; 2 Dipartimento di Scienze Umanistiche, Università Telematica Pegaso, Via Porzio, Centro Direzionale, isola F2, 80143 Naples, Italy; 3 Dipartimento Di Medicina Clinica E Chirurgia, Unit of Endocrinology, Federico II University Medical School of Naples, Via Sergio Pansini 5, 80131 Naples, Italy; 4 School of Medicine, Universidad Católica Santiago de Guayaquil, Av. Pdte. Carlos Julio Arosemena Tola, Guayaquil, 090615 Ecuador; 5 Departamento de Bioquímica Humana, Facultad de Medicina, Universidad de Buenos Aires, C1121ABE Buenos Aires, Argentina; 6 Equipo de Soporte Nutricional, Hospital Británico de Buenos Aires, Buenos Aires, Argentina; 7 Department of Physiology, Faculty of Medicine, University of Colombo, Colombo, Sri Lanka; 8 Institute of Health and Biomedical Innovation, Queensland University of Technology (QUT), Brisbane, Australia; 9 Cattedra Unesco “Educazione Alla Salute E Allo Sviluppo Sostenibile”, University Federico II, Naples, Italy

**Keywords:** Menopause, Obesity, Sleep quality, Sleep disturbances, Chronotype

## Abstract

**Purpose of Review:**

This review primarily provides an overview of the current evidence on chronotype (which reflects an individual’s preference for the timing of sleeping, eating, and activity in a 24-h period) and sleep quality changes in obesity in postmenopausal women. It also explores possible nutritional strategies to manage these changes in this phase of a woman’s life.

**Recent Findings:**

Menopause is a life stage frequently associated with sleep disturbances and changes in circadian rhythms. Sleep disturbances are one of the main symptoms of menopause and are caused by several factors such as hormonal changes, obesity, and melatonin reduction. In addition, the chronotype also changes following menopause. Nutritional strategies are essential because they could help manage menopausal sleep disturbances and circadian misalignment, particularly by tackling obesity and overweight.

**Summary:**

In the management of postmenopausal women, especially those experiencing obesity, careful assessment of sleep disturbances and chronotype and subsequent development of the most appropriate treatment, including nutritional management, should be part of the treatment routine.

## Introduction

A person’s attitude that determines individual circadian preference in behavioral and biological rhythms related to the external light–dark cycle is commonly referred to as chronotype [1]. There are three general categories of chronotypes based on the variants of the behavioral circadian phenotype: morning, evening, and intermediate chronotypes [2]. The morning chronotype (defined as “*lark*”) tends to wake up early and prefers activities at the beginning of the day, while the evening chronotype (defined as “*owl*”) generally wakes up later and prefers its main activity in the late afternoon or evening. The intermediate chronotype occupies an intermediate position between the morning and evening chronotypes [2].

Menopause is a physiological event in a woman’s life characterized by the permanent cessation of ovarian follicular activity, causing an abrupt drop in estrogen levels, resulting in the classic signs and symptoms of menopause [3].

Recent data have demonstrated that the chronotype categories’ prevalence varies considerably between premenopausal and postmenopausal women [4•]. Specifically, in a very recent study, premenopausal women with obesity (*N* = 49) showed a significantly higher likelihood of having an intermediate chronotype, whereas postmenopausal women with obesity (*N* = 72) tended to have a morning chronotype [4•]. Indeed, menopause is characterized by a change in lifestyle (i.e., waking up earlier and eating breakfast earlier) that generally occurs with age [5, 6] while premenopausal women are likely to tend to have a less pronounced morning chronotype, due in part to a more hectic lifestyle and more obligations during the day. In fact, it is stated that with increasing age a progressive trend towards more morning chronotypes is reported [7]. Another factor that could play a role in the change of chronotype category over the years is the fall in estrogen, and the consequent impact on the sleep–wake rhythm [8]. Finally, reactivity to stress, mediated by the action of cortisol, could be an important factor contributing to circadian changes [9].

Sleep disturbances (SD) are common in postmenopausal women, which negatively affects quality of life [10]. In this regard, the 2005 National Institutes of Health State-of-the-Science Conference Statement cites SD as a core symptom of menopause [11]. The etiology for SD in postmenopausal women is still controversial, but the main players seem to be the decrease in estrogen levels [10], weight gain, especially the increase in visceral adiposity [12], and the decrease in melatonin [13].

Although limited data are available on SD and diet in postmenopausal women, overall, they suggest that dietary strategies may be a useful tool in the management of SD in postmenopausal women especially with a concomitant condition of obesity. Indeed, several foods have been associated with improvements or conversely worsening in sleep parameters [14–18]. In addition, circadian rhythms (CR) also seem to be affected by the influence of certain foods, as well as the timing of their introduction [19]. In this context, chrononutrition, a nutritional model based on eating certain foods at specific times of the day according to the rhythms of chronobiology [19], is becoming increasingly popular.

Therefore, the aim of this review is to provide an overview of the current evidence on changes in chronotype and sleep quality in obesity in postmenopausal women. It will also explore nutritional strategies to manage these changes in this stage of a woman’s life.

## Sleep Quality, Obesity, and Menopause

Currently, SD are a common problem in the general population; the prevalence of SD in women varies and increases with age [20]. It ranges from 16 to 42% in premenopausal women, from 39 to 47% in perimenopausal women, and 35 to 60% in postmenopausal women [20], with an even greater risk if obesity being present at the same time [21]. The most common SD in postmenopausal women include insomnia, nocturnal breathing disorders, obstructive sleep apnea (OSA) in particular, restless legs syndrome, periodic limb movement syndrome, depression, and anxiety [10]. The etiology of SD in postmenopausal women is still unknown, but it appears that they are caused by the contribution of several risk factors that commonly occur during menopause, such as decreased estrogen levels and the resulting vasomotor symptoms (VMS), depression, weight gain (especially the increase in visceral adiposity) [10], and a decrease in melatonin levels [22].

The female post reproductive lifespan is characterized by very low estrogen levels, which result exclusively from the peripheral conversion of testosterone to estrogens, as the ovaries no longer have production [3]. The impact of estrogen levels on SD is complex, as estrogens clearly have a wide range of potential effects that impact sleep through multiple mechanisms [23]. Estrogens play a role in the metabolism of norepinephrine, serotonin, and acetylcholine—neurotransmitters that in turn influence sleep control [10]. In fact, estrogens have been reported to shorten sleep latency and increase total sleep time, decrease the frequency of post-sleep awakenings, and decrease cyclical spontaneous arousals [10]. The most common symptoms associated with low estrogen levels are VMS, also called hot flashes (HF) [24]. These symptoms are most common in midlife women and are reported by 75 to 85% of postmenopausal women [24, 25]. Several studies have found an association between VMS and SD in postmenopausal women [26, 27]. Ensrud et al. conducted a randomized trial of 217 healthy postmenopausal women aged 40–60 years with VMS [26]. The women with a higher frequency of moderate to severe HF were more likely to suffer from insomnia, probably due to more frequent disturbed sleep and greater nocturnal wakefulness due to numerous arousals [26]. Recently, Lampio et al. recruited 158 healthy women (107 premenopausal and 51 postmenopausal) in a cross-sectional study to examine sleep quality and its association with night sweats and HF [27]. Postmenopausal women had poorer overall sleep quality, slept more restlessly, and had more nighttime awakenings compared to premenopausal women. SD were mostly associated with night sweats and HF [27].

Overweight and obesity are common in postmenopausal women [28], largely due to changes in reproductive hormone levels [29]. In fact, there is evidence that estrogen depletion affects fat distribution, resulting in increased abdominal fat in postmenopausal women [30–32]. Indeed, estrogens increase lipolysis and affect lipoprotein lipase activity in adipose tissue [33]. Specifically, estradiol may indirectly affect lipolysis by inducing the lipolytic enzyme hormone-sensitive lipase or directly increasing the lipolytic effect of epinephrine [34], such that menopause is associated with an increase in fat mass. It is well known that visceral adipose tissue is an important source of pro-inflammatory adipocytokines such as plasminogen activator inhibitor-1 (PAI-1), interleukin 6 (IL-6), tumor necrosis factor a (TNF-a), and leptin, as well as lower adiponectin levels [35–38]. These pro-inflammatory cytokines could be associated with sleep regulation and classified as “sleep-regulating agents” [12]. Consistent with this, waist circumference (WC), an indirect measure of visceral adipose tissue, has been reported to be associated with SD [39], and these findings have also been confirmed in postmenopausal women. Morfeno-Vicino et al. conducted a study of 463 community-dwelling older Spanish women and reported a significant positive correlation between SD and WC [40]. Among SD in postmenopausal women, OSA was a common finding [40]. In a cross-sectional study, Polesel et al. examined the occurrence of OSA at different stages of the reproductive lifespan such as premenopause, early postmenopause (up to 5 years into menopause), and late postmenopause (> 5 years into menopause) [41]. Similar results were found in a study carried out by Naufel et al. investigating the association between obesity and SD in postmenopausal women [42]. Fifty-three postmenopausal women were enrolled and underwent to anthropometric measurements and full-night polysomnography. As expected, respiratory disturbance index and apnea–hypopnea index values were worsened in women with obesity [42].

Melatonin, a hormone secreted and synthesized by the epiphysis mainly at night under normal light–dark conditions, plays a major role in regulating CR, especially in sleep onset and in sleep maintenance through block arousal mechanism [13]. Melatonin levels decrease with aging after the age of 50 [22]. Melatonin age-related decline is correlated with a decreased melatonin biosynthesis and release by the pineal gland, which is considered due to decreased retinal light perception and the changing nature of the vitreous body, which transmits less light [43]. Thus, the reduction of melatonin levels usually occurs contemporarily to menopause age [44]. In a group of 79 healthy women, Fernandez et al. have evaluated the morning levels of serum melatonin, FSH, LH, prolactin, progesterone, and estradiol. The women were subdivided in three groups by different reproductive stages (fertile stage, perimenopausal, and postmenopausal period). Serum melatonin levels decreased with age, attaining minimum levels in menopause [44]. Indeed, in a prospective study, Toffo et al. showed that the duration of secretion and concentration of melatonin tended to be lower in postmenopausal women (aged 58–71 years) than in perimenopausal [45].

## Chronotype and Menopause

It has been reported that when women approach menopause, changes in CR occur [5, 46, 47]. Most recent evidence has shown that after the menopausal transition there is an evolution towards a more morning chronotype. Specifically, Gomez Santos et al. in their study reported that postmenopausal (*N* = 50) women tended to be more a morning chronotype in behavior and physiology compared with premenopausal women (*N* = 127) [5]. In fact, postmenopausal women used to wake up and eat breakfast earlier in the morning than premenopausal women, tending towards an increasingly morning chronotype as they got older [5, 6]. Conversely, premenopausal women are likely to tend towards an evening chronotype as they are partly related to a more hectic lifestyle and more commitments during the day; it is known that the chronotype is partly dependent on environmental factors [48–50]. Furthermore, the chronotype is linked to age: children are generally morning chronotypes [7]. As they grow older, they become more and more evening chronotypes, until a peak around the age of 20, after which they become progressively more morning chronotypes again. In addition, a gender difference in the chronotype has been reported [51]; as is well known, females reach puberty earlier than males and this is also reflected in the chronotype: young females develop an evening chronotype earlier than young males. This gender difference disappears around the age of 50, which coincides with the average age of menopause [7]. This could be due to a combination of less regular social and light schedules or a less robust circadian system in later life [52, 53].

Since menopause involves well-known hormonal changes, it is possible to assume that these also play a part in the change in chronotype categories over the years [8]. Indeed, the concentration and timing of release of many hormones are age-dependent [54]. In addition, estrogen also has an antidepressant effect [55]. With less estrogen, postmenopausal women may experience higher body temperature, poorer sleep quality, and lower mood [56]. The sleep–wake cycle also changes with age and loses its constancy. Postmenopausal women feel tired earlier and wake up earlier in the morning, resulting in less sleep overall [5]. Consistent with these data, Gomez Santos et al. found that postmenopausal women slept 10% fewer hours than premenopausal women [5]. Studies of sleep quality during the menopausal transition and after menopause found that lower estradiol levels were associated with nocturnal awakenings [24, 57]. Results from the Study of Women’s Health Across the Nation (SWAN) indicated that lower estradiol levels and higher FSH were associated with difficulty falling asleep and staying asleep [24]. Furthermore, these findings were confirmed in later studies of polysomnographic sleep in a subgroup of SWAN participants [57]. Indeed, this study found increased beta-EEG (electroencephalography) power in NREM (non-rapid eye movement) sleep in postmenopausal women, suggesting that the menopausal transition is associated with physiological overexcitation during sleep, independent of self-reported HF [57].

An association between sleep changes and cortisol with aging has been reported [58, 59]. Cortisol levels at baseline and circadian nadir are elevated in healthy elderly people compared to healthy young people, while the circadian amplitude decreases with age in both women and men [58]. In adults, cortisol levels are lower in premenopausal women than in men of the same age. After menopause, there are no significant gender differences in total plasma cortisol levels [58]. Thus, these data could explain the tendency towards a morning chronotype in postmenopausal women. Cortisol is the stress hormone [60] and furthermore, gender differences in stress response have been suggested [61]. Older women show greater effects on sleep parameters than men of the same age during mild stress, specifically an increase in sleep latency, a decrease in time in bed, a decrease in total sleep time, and an increase in total time awake [61]. In this respect, it has recently been shown that the circadian pacemaker in the central nervous system is anatomically and functionally connected to the paraventricular neurons that control the release of corticotropin-releasing hormone and adrenocorticotropic hormone [62], which are known to be activated by stress. Considering this evidence, stress reactivity could be an important factor contributing to age-related sleep quality and circadian changes.

## Nutritional Approach to Women with Menopause and Obesity and Sleep Quality/Chronotype

The associations between various lifestyle factors including dietary practices with sleep quality among postmenopausal women have been identified. Observational studies report an association between dietary intake and menopausal symptoms including SD [63–65]. High intake of fruits and vegetables, whole grains, and unprocessed foods has protective effects whereas high sugar and saturated fats have adverse relationships [66••]. A large cohort study of over 5 years of follow-up of midlife Mexican women reported that in a fully adjusted models, those who consumed least amount of fruits and vegetable and highest amount of modern Mexican food patterns are significantly associated with poor quality of sleep [67]. A cross-sectional study of 769 postmenopausal women showed that a lower diet quality is associated with the lowest quality of sleep, as well as high fat consumption is associated with restless sleep [68]. The National Health and Nutrition Examination Survey (*N* = 1783) showed that postmenopausal women with more sugar intake and lack phosphorus and zinc are associated with short sleep duration [69]. Finally, a very recent cross-sectional study in 100 postmenopausal women with obesity showed that legume consumption was associated with lower menopausal symptom severity (evaluated with the Menopausal Rating Scale) while extra virgin olive oil consumption was associated with lower psychological symptoms [70]. In the same study, women with the higher severity of menopausal symptoms also had the lower adherence to the Mediterranean diet [70]. In addition to dietary habits, obesity (BMI > 30 kg/m^2^) and abdominal obesity are associated with SD in postmenopausal women [42]. It is believed that “sleep-regulatory substances” produced from adipocytes might be associated with SD in postmenopausal women [46]. Moreover, obesity especially abdominal obesity is strongly associated with OSA syndrome among postmenopausal women [41]. An adjusted model for age, sex, and BMI showed that a higher daily isoflavone intake from food was significantly associated with better sleep duration and higher sleep quality in 1076 Japanese adults [71]. Hachul et al. supplemented 80 mg of isoflavones daily for 4 months in a placebo-controlled, double-blinded study and reported a significant improvement in insomnia in postmenopausal women (*N* = 38) compared to placebo [14]. A significant relief in SD was observed by adding nutraceutical combination (*Lactobacillus sporogenes* 109 spores, *Magnolia officinalis* extract 50 mg, *Vitex agnus-castus* extract 40 mg, and vitamin D 35 μg) to soy isoflavones for a 12-month period in a randomized controlled trial (RCT) among 180 postmenopausal women [72]. In another intervention, adding calcium (141 mg) and magnesium (50 mg) in addition to soya isoflavones, probiotics, *Magnolia*, and vitamin D reported an improvement in SD [73]. A systematic review and meta-analysis of 62 RCT showed that phytoestrogen supplements have modest but significant reduction in HF [74]. Since HF are strongly associated with insomnia [75], phytoestrogen may have indirect effect on controlling SD during the menopause period. Moreover, phytoestrogens are safe and might be protective against breast cancers [76]. In a randomized, double-blind, placebo-controlled trial, 60 postmenopausal women on supplementing 1000 mg of omega-3 fatty acid daily for 3 months showed a significant improvement in sleep problems [77]. Combination of resveratrol, tryptophan, glycine, and vitamin E is a potential nutraceutical useful in the prevention of SD in postmenopausal women [78]. In a RCT on 35 older people, which compared the high dose of tryptophan (120 mg/day) to low dose of tryptophan (45 mg/day), those who consumed high tryptophan cereal meals showed an improvement of several sleep index including increased sleep efficiency, actual sleep time, and sleep latency [79]. Exercises, in addition to dietary modifications, are beneficial and should be coupled for best outcomes. In a group of 106 late peri- and postmenopausal, sedentary women, a 12-week, individual, facility-based moderate-intensity aerobic exercise program showed an improvement in insomnia symptoms, and subjective sleep quality [80].

Since it has been reported that postmenopausal women with an evening chronotype, escaping the “physiological” propensity for a morning chronotype, have higher health risks, such as more obesity or cardiometabolic diseases [46, 81], new nutritional approaches that correct circadian alterations should be developed. Chrononutrition is a new area of research that investigates the effects of diet (particularly timing and nutrients) on CR [19]. In this context, foods and macronutrients at specific times of day have been shown to influence circadian oscillations in clock genes [19]. For instance, it has also been documented that the fat consumed can influence the genes involved in the CR through epigenetic modifications [82]. Specifically, consumption of monounsaturated fatty acids (MUFA) and polyunsaturated fatty acids (PUFA) may affect the methylation levels of the cytosine of the CpG islands in the CLOCK promoter, thereby regulating gene expression of the circadian clock, inhibiting it based on CLOCK polymorphism. High consumption of MUFA and olive oil is negatively associated with CpG methylation levels of CLOCK, whereas that of PUFA is positively associated. Therefore, CpG methylation levels of CLOCK together with other genes could be used as markers for weight loss [82].

Excessive carbohydrate consumption in the evening leads to a rise in blood glucose levels the next morning [83]. Some studies have investigated whether the glycemic index (GI) of meals eaten at different times of the day affects insulin levels and postprandial glucose response. A randomized crossover study in 6 healthy subjects showed that a meal with a high GI in the evening elicited a stronger glucose and insulin response than in the morning [83]. Another recent cross-sectional study in 112 women with polycystic ovary syndrome showed that evening chronotype was associated with a most severe insulin resistance and unhealthiest eating habits like higher intake of total and simple carbohydrates [84]. The differences in glucose sensitivity between morning and evening are due to the circadian fluctuations of insulin, which peaks during the day and then decreases its sensitivity in the evening [85]. In humans, glucose metabolism is subject to a CR with a diurnal variation in glucose tolerance that typically peaks during the day [86, 87]. This glucose rhythm is related to food intake, with peak glucose tolerance accompanying food intake (during the day) and decreasing with fasting during the dark night hours [86]. Interestingly, hormones such as insulin and cortisol, which are involved in glucose metabolism, also show a circadian oscillation [88]. Thus, sensitivity and insulin secretion are closely regulated by chronobiological rhythms, which has strong implications for glucose metabolism [88]. Furthermore, perturbations in glycemia levels at nighttime are reduced with meals high in protein compared to meals high in carbohydrate [89]. Also, it has been observed that high-protein diets lead to greater reductions in total energy intake, body weight, and fat mass while preserving lean body mass compared to a normal protein diet [90]. Since some typical menopausal factors lead to alterations in glucose metabolism in these women [91], giving special attention to the timing of carbohydrate intake during the day could be a useful strategy. Indeed, the changes in body fat distribution, the production of inflammatory cytokines by adipose tissue, and the increased androgenic status of postmenopausal women are associated with several alterations in glucose metabolism such as reduced insulin sensitivity and insulin resistance [91]. In addition, Oseguera-Castro et al. recently investigated the effects of dietary fiber intake (21 days/45 g serving) on CR modulation in young adults. Biscuits containing 3 different types of fiber were consumed: (1) isolated fiber from coffee grounds, (2) a combination of coffee grounds and fructooligosaccharides, and (3) fiber-free biscuits [92]. The biscuits with isolated fiber and with fructooligosaccharides decreased evening chronotypes, improved sleep quality and length, and enhanced chronodisruption associated with short-chain fatty acid production in the colon [92].

Other popular foods capable of affecting the circadian cycle include caffeine which has been shown to be able to affect the phase of gene expression of the peripheral circadian clock tissues of mice, and its use is able to alter the CR after jet lag [93]. High sodium chloride consumption may also affect the circadian cycle [94]. High salt consumption over 2 weeks causes a change in the circadian cycle in the kidneys, liver, and lungs [94]. Alcohol appears to disrupt molecular, endocrine, and behavioral CR in humans and other animals [95]. Another group of compounds that may affect CR are polyphenols, including those of tea [96]. These polyphenols have been shown to ameliorate the metabolic syndrome through mechanisms related to circadian clock, restore nucleus genes, mitigate attenuated diurnal variation, and control the clock of genes induced by constant darkness, thereby reversing intolerance to glucose and insulin. This shows that there is an oscillator that can be triggered by the tea polyphenol [96].

## Conclusion

**Fig. 1:**
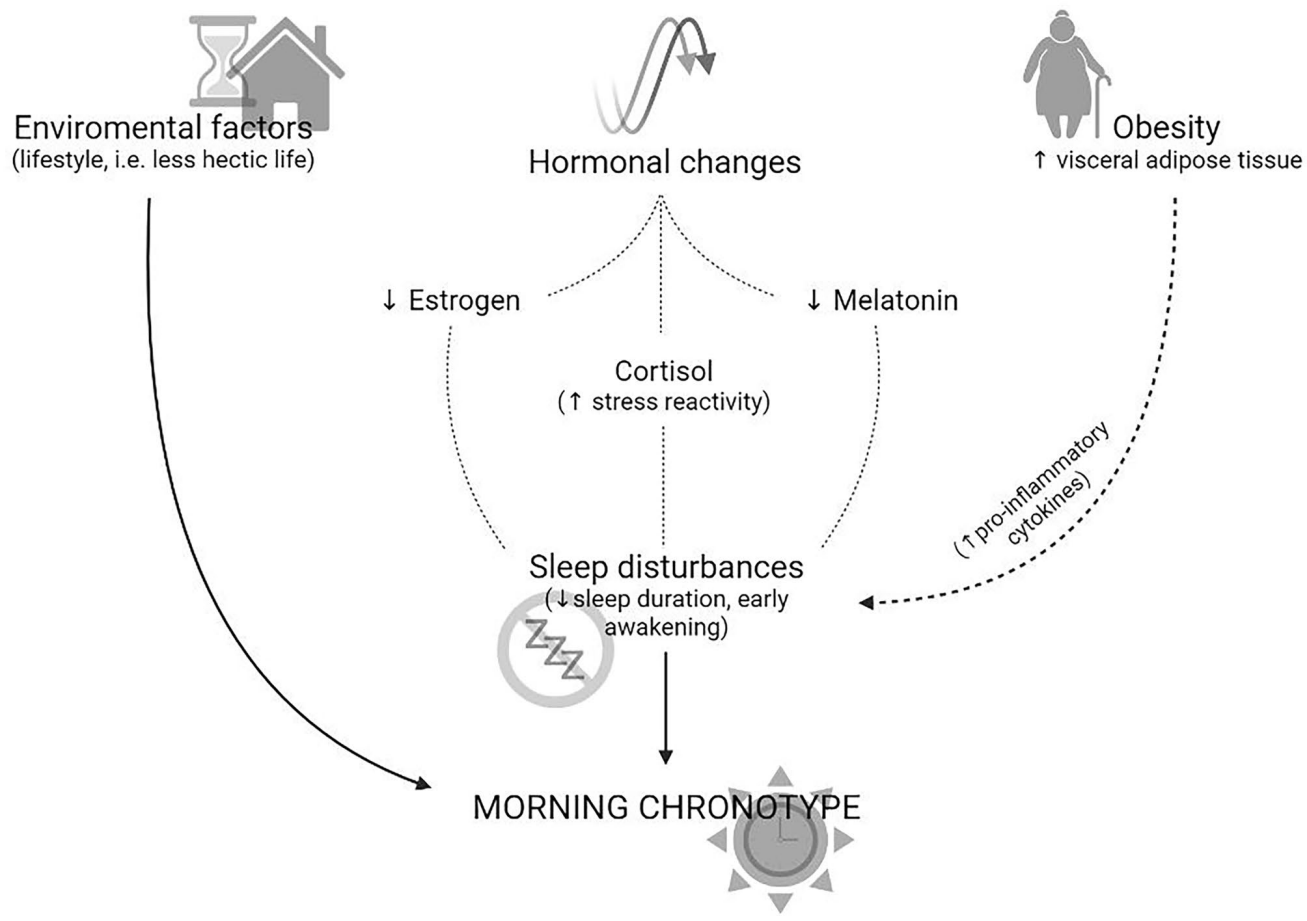
Pathophysiology of changes in sleep quality and chronotype in postmenopausal women

The menopause is characterized by an increase in SD and a tendency towards a morning chronotype (Fig. 1). The mechanisms underlying the higher prevalence of SD in menopausal women compared to premenopausal women are many and still under investigation. Similarly, research on chronotype and its impact on health in this specific setting of subjects is recent and needs further study. In addition, based on the latest evidence, it seems appropriate to evaluate specific nutritional approaches for these women taking also into account SD and individual chronotype. Thus, in the treatment of menopausal women, especially those living with a condition of obesity, a careful assessment of SD and chronotype and the subsequent development of the most appropriate management, including nutritional management, should be part of the treatment routine.
